# Cabozantinib, Vandetanib, Pralsetinib and Selpercatinib as Treatment for Progressed Medullary Thyroid Cancer with a Main Focus on Hypertension as Adverse Effect

**DOI:** 10.3390/ijms24032312

**Published:** 2023-01-24

**Authors:** Linnea Højer Wang, Markus Wehland, Petra M. Wise, Manfred Infanger, Daniela Grimm, Michael C. Kreissl

**Affiliations:** 1 Department of Biomedicine, Aarhus University, Ole Worms Allé 4, 8000 Aarhus, Denmark; 2 Department of Microgravity and Translational Regenerative Medicine, Otto von Guericke University, Universitätsplatz 2, 39106 Magdeburg, Germany; 3 Clinic for Plastic, Aesthetic and Hand Surgery, Otto von Guericke University, Leipziger Str. 44, 39120 Magdeburg, Germany; 4 Department of Radiology and Nuclear Medicine, Division of Nuclear Medicine, Otto von Guericke University, Leipziger Str. 44, 39120 Magdeburg, Germany

**Keywords:** medullary thyroid cancer, tyrosine kinase inhibitors, cabozantinib, vandetanib, pralsetinib, selpercatinib, adverse effects, hypertension, clinical trials

## Abstract

This manuscript investigates cabozantinib, vandetanib, pralsetinib, and selpercatinib, four tyrosine kinase inhibitors (TKIs), which are used to treat advanced and/or metastatic medullary thyroid cancer (MTC). Data on efficacy and safety are presented with the main focus on treatment-related hypertension, a well-known adverse effect (AE) of these TKIs. Taken together, TKI-induced hypertension is rarely a dose-limiting side effect. However, with increasing survival times of patients under treatment, hypertension-associated complications can be expected to be on the rise without proper medication.

## 1. Introduction

### 1.1. Thyroid Cancer

Thyroid cancer (TC) is the most common type of endocrine malignancy and accounted for 3% of all new cancer cases in 2020 [1], with more than 85% of cases being papillary thyroid cancer. The incidence increases, which has been attributed at least in part to improved detection through diagnostic procedures [2]. TC can be divided into different histological types: differentiated thyroid cancer (DTC), where the cancer growth evolves in the follicular cells and which can be coarsely divided into the by far most commonly found papillary cancer, follicular thyroid cancer, and the less common poorly differentiated thyroid cancer. Furthermore, there is undifferentiated thyroid cancer (anaplastic TC) arising from follicular cells as well, which is also rare, but due to complete dedifferentiation, this type of cancer is very aggressive and almost always fatal [1,3].

The third type, which is the topic of this review, medullary thyroid cancer (MTC), is rare, only constituting 3–4% of all TC types. It is derived from the parafollicular cells (C cells) of the thyroid gland and, therefore, biologically distinct from DTC, constituting a basically different tumor entity. MTC appears as a sporadic (75%) and an inherited form (25%), often with the germ-line-mutation of the proto-oncogene rearranged during transfection (RET) coding for a receptor tyrosine kinase (RTK) [4,5]. First-line therapy and the only curable treatment for MTC is a thyroidectomy combined with cervical lymph node dissection [6,7,8]. However, in patients with distant metastasis and progression, systemic treatment is crucial to slow tumor growth and improve quality of life. C-cells produce calcitonin, do not take up (radio-) iodine, and are not influenced by thyroid-stimulating hormone (TSH). Therefore, radioiodine treatment and TSH-suppressive therapy are not options [9,10,11]. Instead, tyrosine kinase inhibitors (TKIs) are used as a treatment to inhibit oncogenic pathways [12]. The first-line options for patients with advanced and progressive MTC are either vandetanib or cabozantinib, which are also approved in this indication. Two new targeted drugs, selpercatinib and pralsetinib, have been recently introduced, and the good results achieved in the respective clinical trial will have an impact on the treatment scheme. A common side effect of TKIs is hypertension which needs to be kept under control to avoid cardiovascular events and a decrease in quality of life [13,14].

### 1.2. Tyrosine Kinase Receptors

**Figure 1:**
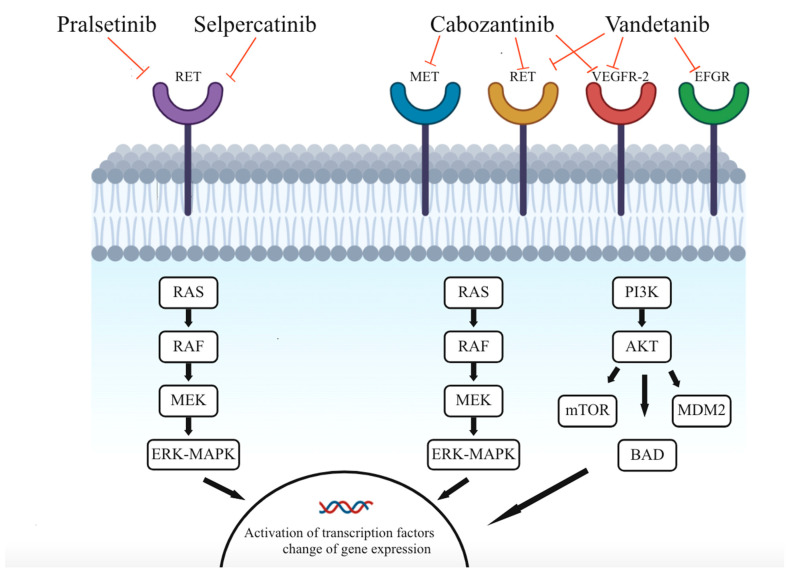
The mechanism of pralsetinib, selpercatinib, cabozantinib, and vandetanib inhibiting different receptors and their respective intracellular cascades. Only receptors inhibited by cabozantinib being investigated in the EXAM study are mentioned. RET: Rearranged during transfection. MET: Tyrosine-protein kinase met or hepatocyte growth factor receptor. VEGFR-2: Vascular epithelial growth factor 2. EFGR: Epidermal growth factor receptor. RAS: Rat sarcoma. RAF: Rapidly accelerated fibrosarcoma. MEK: Mitogen-activated protein kinase kinase-1. ERK: Extracellular regulated kinase. MAPK: Mitogen-activated protein kinase. PI3K: Phosphoinositide 3 kinases. AKT: Protein kinase B. mTOR: Mammalian target of rapamycin. MDM2: Mouse double minute 2. BAD: BCL2 associated agonist of cell death. The figure was created in Miro.

TKIs inhibit various cell surface kinases called RTK. The binding of its ligand induces dimerization leading to autophosphorylation of their cytoplasmic domains, causing a change in conformation and thereby activation of the intracellular cascades RAS/MAPK and PI3K/mTOR pathway (Figure 1). This induces changes in transcription factors leading to an extended expression of genes encoding for cell proliferation and growth, angiogenesis, and lymphangiogenesis. Blockage of these intracellular phosphorylation cascades is of great interest because they show a suppressing oncogenic effect and can be induced by TKIs [15,16].

Several oncogenic pathways are known to be present in MTC [14]. An example is the RTK mesenchymal-epithelial transition factor (MET) situated in the epithelial cells of multiple organs and becomes activated when the ligand hepatocyte growth factor (HGF) binds. The intracellular pathways PI3K/mTOR and RAS/MAPK are activated and signal cell survival and cell proliferation. Interference with such cascades can lead to the progression and metastatic spread of MTC [17]. The growth factor (GF) vascular endothelial GF (VEGF) is also of great importance regarding tumorigenesis. Overexpression of VEGF and its corresponding receptor VEGFR-2 is seen during tumorigenesis, partially due to the para- and autocrine secretion from the tumor cell itself. VEGFR-2 is an RTK expressed on epithelial cells in the blood vessels. When activated with VEGF-A, it stimulates vascular permeability and tumor-associated angiogenesis (formation of new blood vessels from pre-existing vessels) and plays a key role in malignant tumor initiation and proliferation [18,19]. VEGF-A also regulates the development of new lymphatic vessels when binding to VEGFR-3 on lymphatic endothelial cells, a stimulation leading to lymphangiogenesis in tumors and metastasis [16]. The RET oncogene encodes for an RTK, constitutive activation caused by fusions or mutations that lead to the development of cancer. Mutations in this gene are present in 55.8% of all sporadic MTC at initial diagnosis and in virtually all hereditary MTC, making the mechanism of RET important. It causes a gain-of-function kinase activity of the pathway RAS/MAPK, often due to point mutation or gene fusion which exerts a crucial role in MTC oncogenesis [3,20].

### 1.3. Multikinase Inhibitors

Cabozantinib inhibits RTK such as MET, RET, VEGFR-1, -2, and -3, tyrosine-protein kinase KIT (KIT), TRKB (tropomyosin receptor kinase B), fms-like tyrosine kinase 3 (FLT-3), tyrosine-protein kinase receptor UFO (AXL), and angiopoietin-1 receptor (TIE-2) by blocking the intracellular phosphorylation cascades, thus suppressing oncogenesis [21]. Inhibiting VEGFR alone entails an overexpression of MET, which leads to a promotion of tumor growth, angiogenesis, and metastasis. It, therefore, is a logical step to use a drug, like cabozantinib, that inhibits both VEGFR and MET at the same time [22].

Vandetanib inhibits RET, VEGFR-2, and epidermal growth factor receptors (EGFR). EGFR is a receptor leading to cell growth and contributes to RET kinase activation. Due to the high prevalence of RET mutations in MTC, the inhibition of EGFR may indirectly provoke cancer activity [16].

### 1.4. Selective Inhibitors

RET mutations are numerous in inherited and sporadic MTC, making selective RET inhibitors attractive as treatment options. The toxicity profile of TKIs with a broad spectrum of adverse effects (AEs) largely cannot be attributed to the inhibition of RET. It can be expected that RET inhibitors have considerably lower toxicity [23].

Selpercatinib is a selective RET inhibitor with potency against various mutations and shows a marked antitumor activity [24,25].

Pralsetinib is also a treatment for RET-altered MTC patients [26]. Compared to the conventional treatment with vandetanib and cabozantinib, it is 8–28 times more potent against wild-type RET and other RET alterations [27]. Table 1 gives an overview of the TKIs discussed in this review.

### 1.5. Hypertension

Hypertension is a rising problem and remains the foremost risk factor for death in the general population [33]. The pathophysiology of elevated blood pressure (BP) is very complex due to its secondary causes. High BP can induce cardiovascular and renal diseases and stroke, leading to organ failure. Hypertension is defined as systolic blood pressure ≥140 mmHg and diastolic blood pressure ≥90 mmHg. Values above this threshold need treatment, but it would unequivocally bring more benefits than harm despite the risks following antihypertensive treatment. To simplify how to treat patients with hypertension, BP measurements are put into cut-off values and are correspondingly divided into categories depending on their severity (Table 2) [34,35].

Management of hypertension can be non-pharmacological, which includes lifestyle changes: reduced alcohol and salt consumption, a healthy diet, smoking cessation, and increased regular physical activity [36]. Non-pharmacological treatment is most often for patients in the “high normal” group, where no drug-induced treatment is offered. The same accounts for grade 1 hypertension, but if BP does not decrease within 6 months, the newest studies show that pharmacological treatment is needed to reduce the risk for cardiovascular disease [37,38].

Patients with grade 2 and 3 hypertension should immediately start antihypertensive therapy while making lifestyle changes as well. For the antihypertensive therapy, five drugs are being recommended: Angiotensin-converting enzyme (ACE)-inhibitors, diuretics, angiotensin receptor blockers (ARBs), calcium channel blockers (CCB), and beta-adrenoceptor antagonists (BAAs). They all have different contraindications and preferential use in specific conditions [34,39]. ACE inhibitors and BAAs are preferred in patients with cardiac dysfunction or risk of heart failure. Diuretics are commonly used but carry the risk of electrolyte loss and QT prolongation. Therefore, they should be used with caution [40].

### 1.6. Mechanism of Hypertension as an Adverse Effect

Common to all four drugs is hypertension as a frequent AE. There is a well-known association between hypertension with CVD [37,38,41]. Especially due to the long periods of time on systemic treatment using TKIs or selective inhibitors, an increase in risk for cardiovascular events cannot be excluded in patients with advanced MTC.

**Figure 2:**
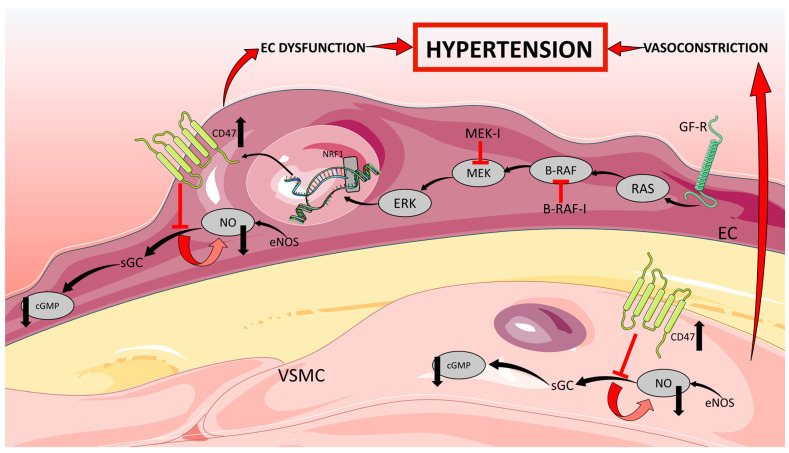
A possible mechanism for BRAF- and MEK-inhibitor-induced hypertension. EC: Endothelial cell; VSMC: Vascular smooth muscle cell; BRAF-I: BRAF-inhibitor; MEK-I: MEK-inhibitor; RAS: NRF1: Nuclear respiratory factor 1; eNOS: Endothelial NO synthase; NO; Nitric oxide; cGC: Soluble guanylate cyclase; cGMP: Cyclic guanosine monophosphate; CD47: Cluster of differentiation 47. Modified from [51]. Some elements of this figure were taken from the smart Servier Medical Art library (https://smart.servier.com/, accessed on 24 November 2022) published under the creative commons Attribution 3.0 Unported (CC BY 3.0) license (https://creativecommons.org/licenses/by/3.0/, accessed on 24 November 2022).

Therefore, the four types of TKIs have all been investigated in relation to the severity of the AE of hypertension, but the underlying mechanism is still unclear. Hypertension mainly seems to be ascribed to the inhibition of VEGF [42,43]. Normally, nitric oxide (NO) and prostaglandins (PGI2) are secreted from endothelial cells in the blood vessels, both causing vasodilation (Figure 2). Experimental data of TKI targeting VEGFR, like cabozantinib and vandetanib, show that inhibition of VEGF decreases NO and PGI2 secretion, which increases the total peripheral resistance (TPR) and thereby increasing BP defined by Ohm’s law (mean arterial pressure (MAP) = TPR × cardiac output (CO)). This could be a possible explanation for VEGF-induced hypertension [22,44,45]. An increased expression of vasoconstrictor endothelin-1 (ET-1) is another cofactor and may contribute to hypertension. Furthermore, VEGF provides a survival signal for endothelial cells, and within the short time after the anti-angiogenic therapy had begun, apoptosis of these cells had been detected, a process referred to as rarefaction. This theory is based on human studies demonstrating fewer dermal capillaries and dilatory responses after achieving treatment. Fewer capillaries give a smaller volume and decreased space for blood which leads to a rise in resistance and, thereby, a higher BP (Poiseuille’s law).

The exact role of rarefaction in hypertension development and maintenance is questionable. Inhibition of VEGF also leads to dysregulation of the renal vascularity, which may alone lead to elevated BP or cause glomerular ischemia and activation of the renin-angiotensin-aldosterone (RAAS) pathway [46], mediating a systemic increase in BP. An increase in arterial stiffness due to VEGF inhibition is also observed, resulting in decreased compliance and, thus, hypertension [47].

The underlying mechanisms for RET-inhibitor-associated hypertension are still not clarified. RET kinase inhibitors like pralsetinib and selpercatinib increase hypertension by upregulation of CD47 (integrin-associated protein), downregulation of cyclic guanosine monophosphate (cGMP), and reduction of nitric oxide (NO) [48]. Thrombospondin-1 and its receptor CD47 are involved in the acute physiological regulation of blood pressure and show a vasopressor activity to maintain global hemodynamics under stress [49]. CD47 is important for the NO-stimulated vascular responses by thrombospondin-1 [50]. In addition, CD 47 is involved in inducing dysfunction of the endothelium [51]. CD47 inhibits the NO/cGMP) signaling pathway, and thus, it leads to changes in blood pressure and vascular tone [51].

ERK plays an important role in the transcriptional activation of CD47 [52]. RET inhibition results in a rebound ERK activation, which is also observed in cases of BRAF/MEK inhibition [48].

Taken together, the increase in CD47 seems to be responsible for the development of RET inhibitor-associated hypertension.

## 2. Results

### 2.1. Efficacy of Systemic Treatment with TKIs and Selective Inhibitors

Tumor assessments in most trials were categorized by using Response Evaluation Criteria in Solid Tumors v1.0 (RECIST) performed at screening prior to the study and every 12 weeks until progression or withdrawal. The efficiency of the different treatments was estimated by the primary endpoint of an objective response rate (ORR), which includes complete and partial responses from the medication defined by RECIST criteria (Table 3).

Tumor regressions were seen in nearly all (RET-mutated) patients receiving selpercatinib (LIBRETTO-001 trial), regardless of the kind of RET mutation or whether the participant had or had not received previous TKI treatment with cabozantinib, vandetanib, or both.

The LIBRETTO-001 trial showed an ORR of 75% (hazard ratio (HR): 0.73; 95%CI: 0.62–0.82; *p* < 0.05) and a progression-free survival (PFS) median of 23.6 months [22,24]. In a small phase II trial (LIBRETTO-321) in a Chinese population with RET-mutant MTC and RET fusion-positive DTC, the objective ORR was found to be 57.7% (95%CI, 36.9–76.6) [53]. Data from the LIBRETTO-531 trial, a phase III study comparing selpercatinib with standard of care TKI treatment [54], will likely be published soon.

Very promising results could be obtained similarly using pralsetinib (ARROW trial) in (RET-mutated) patients exhibiting an overall response rate of 71% (95%CI: 48–89; *p* < 0.05) in patients without previous systemic treatment [24,26,55].

Cabozantinib (EXAM trial) revealed an objective response rate of 28% vs. 0% (HR: 0.28; 95%CI: 0.19–0.40; *p* < 0.001) and a PFS of 11.2 months. Comparing placebo to a subgroup of patients with RET M918T mutation, a significant difference is seen (*p* < 0.03) in overall survival (OS), and they may experience a better treatment benefit [56]. Since the M918T mutation carries a greater risk of aggressive MTC compared to other mutations, these findings are noticeable [57].

Vandetanib showed better results with a higher objective response rate of 45% as compared to placebo with 13% (odds ratio (OR): 5.48; 95%CI: 2.99–10.79; *p* < 0.001) and a PFS of 30.5 months, although these results may partly account for the different inclusion criteria compared to the EXAM trial [30,58]. Other studies of vandetanib also demonstrate a lower PFS at 17 months which supports this statement [59]. All four studies showed a statistically significant advantage *(p <* 0.05) for treatment, but no TKI has induced a significant improvement of OS [60], which can mainly be attributed to the study design. Several studies have drawn this conclusion as well [7,28,61,62].

The study investigating vandetanib (ZETA trial) presented a dose reduction within 35% of the cases and 79% of the patients getting cabozantinib (EXAM) due to AEs of grade 3 or 4. Discontinuation was needed in 12% and 16% of the cases, respectively. Compared to the pralsetinib trial (ARROW trial), where only 4% of the patients discontinued and 0.6% discontinued by selpercatinib (LIBRETTO trial) application [25], selpercatinib led to drug reduction in 31% of the cases (Table 4). Hypertension was observed in all studies, among other AEs. Selpercatinib resulted in hypertension in 21% of the patients, pralsetinib in 17%, cabozantinib in 8%, and vandetanib in 9%, all being of grade 3 or higher [24,25,26,28,30,55] (Table 4).

Hypertension is not the most common or severe AE of TKI therapy due to the well-established treatment options and extensive knowledge about hypertension. It is easy to detect and treat and only seldom the reason for discontinuation, as mentioned in the study of selpercatinib (LIBRETTO trial) and cabozantinib (EXAM trial) [25,28]. The major difference in therapy changes among the four drugs is due to other common AEs like diarrhea, rash, and nausea [60]. Table 5 underlines that such AEs are more common after the application of vandetanib or cabozantinib compared to the two RET-selective drugs.

### 2.2. Clinical Trials

There is a continued need for validation of the four TKIs as a treatment option for patients suffering from MTC. Clinical trials listed in Table 6 are investigating these drugs to find the optimal therapy.

Completed studies (Table 7) are just as important because today’s medication depends on them.

### 2.3. Therapeutic Management of TKI-induced Hypertension

The management of TKI-induced and RET inhibitor-associated AEs is of great importance to improve the quality of life and avoid discontinuation, which can lead to a rapid increase in cancer growth [44,64].

When using antihypertensive drugs, possible drug-drug interactions must be encountered. The enzyme cytochrome P450 3A4 is involved in the metabolism of cabozantinib. The CCB diltiazem interferes as a substrate and should be avoided to not exceed the preferred concentration of the drug in plasma [65,66]. A classic choice of antihypertensive treatment would be the other first-line drugs (Table 8): ACE inhibitors, ARBs, diuretics, or BAA [44,67,68]. Diuretics often imply electrolyte depletion, and with diarrhea, nausea, and vomiting as frequent AEs, this option should be excluded to prevent dehydration. Vandetanib, selpercatinib, and cabozantinib are associated with prolonged QT-times (QT-intervals) on ECG. Therefore, the use of diuretics is a possibly fatal combination [24,25,28,30,66]. Several reports emphasize that angiotensin II stimulates the growth and progression of cancer cells. A study with the ACE inhibitor losartan supports these findings by stimulating a proapoptotic pathway reducing tumor growth and enhancing tumor cell necrosis. Therefore, this class of drugs should be the first choice in the antihypertensive treatment of cancer patients. However, this observation has not been made in MTC but in several other types of cancer [69,70,71].

In theory, VEGF receptor inhibition is one of the main reasons for hypertension and is of great importance in treating hypertension. The blockage of VEGF leads, most importantly, to the suppression of NO production, which can be prevented by giving nebivolol, a BAA-inducing NO signaling [66]. NO donors have not only shown good results as a treatment but also with a preventive effect for drug-induced hypertension. Further investigations are needed to draw a final conclusion [43,72].

## 3. Discussion

Due to the different inclusion criteria and a very rare tumor entity, no direct comparison between vandetanib (ZETA trial) and cabozantinib (EXAM study) has been made. All mentioned studies (Table 3), apart from the ZETA trial, recruit(ed) patients based on RECIST criteria. Recruitment for the ZETA study only required a tumor sample, so the analyses of the ZETA trial may be subject to confounding due to the different baseline characteristics [58]. Patients with less progressive cancer can participate in the study, and this fact attributes to the greater PFS of 30.5 vs. 19.3 months in the placebo group. When comparing the placebo group with the placebo group of the EXAM trial (PFS of 4 months), no major difference should be seen, which emphasizes the limited data from the ZETA trial. Therefore, another sub-analysis was performed from the ZETA trial data, only including patients with progressive and symptomatic MTC, which should be more comparable to RECIST criteria. This subgroup exhibited a PFS of 21.4 months and a placebo of 8.4 months which are more similar to the results of cabozantinib [63], but the subgroup is smaller and not size comparable to the EXAM study.

Real World Data has compared the efficiency of cabozantinib and vandetanib based on other retrospective results from patient records. The median PFS in vandetanib was 12 months, and 9 months in cabozantinib [14,59]. This data is derived from patients being treated in four German tertiary care centers, which decreases the validity. However, more remarkable is that the patients suffering from worse AEs also responded better to the cancer therapy, a tendency seen in more studies. In long-term treated patients with vandetanib, a significant association between the presence of AEs and response to the treatment is seen. This observation should, first and foremost, induce continuation despite a certain degree of AEs. Another frequent tendency was that the best responses occurred in younger patients because they adapted better. This opens up the discussion about whether the treatment should be induced as early as possible. Clarification on this issue still needs to be investigated [73,74].

The general poor prognosis, when diagnosed with progressed MTC, can be assigned to the development of therapy resistance. Mutations in the kinase domain can prevent the binding and effect of TKIs. This mechanism is expressed in multiple patients becoming less responsive after TKI intake for a certain time [75]. Despite this mechanism, pralsetinib and selpercatinib show improved ORRs in a collective of patients with a RET mutation. When comparing the four drugs with each other, the major differences not only rely on ORRs, but also on the different patient collectives, the percentage of dose-reduction, interruptions, and discontinuation (Table 4). Based on these results, selpercatinib is favored as a first-line treatment due to its greatest ORR, lowest dose reductions, interruptions, and discontinuation, and similar results are obtained for patients treated with pralsetinib. One might think that these bettering results are due to the lack of VEGR inhibition, but when comparing the incidence of hypertension, no major differences can be seen (Table 5) and thus cannot explain the superior results from selpercatinib and pralsetinib. This underlines the minor impact of hypertension in TKI treatment, probably due to good management.

Both studies of pralsetinib (NCT03037385) and selpercatinib (NCT04280081) comprise a smaller patient group with 29 and 88 cases, respectively, compared to 231 and 219 patients in vandetanib and cabozantinib trials (Table 3); neither are they compared to placebo making it difficult to distinguish between the placebo effect and the natural risk of high BP (or other AEs) occurring despite the treatment. This leads to an overestimated percentage of AEs and cannot explain the lowered need for discontinuation. Another weakness is the lack of long-term data since the approval of pralsetinib and selpercatinib are based on evidence from phase 1 and 2 studies [23].

A study with patients receiving a lower vandetanib dose of 100 mg/day displayed less hypertension as compared to 300 mg. The same results were obtained in a study with cabozantinib, where fewer AEs occurred with a dose of 60 mg/day compared to 140 mg/day (63% vs. 72%). The same ORR was seen in both doses [76], clearly that the therapy should be conducted with a lower dose.

The amount and severity of AEs are important because hypertension can cause CVD, which accounts for approximately 21% of deaths in thyroid cancer in general [77], and should be the real concern when trying to further reduce all-cause mortality (40). Antihypertensive drugs are used to keep the hypertensive AEs under control, but these drugs alone also entail AEs like headache, edema, and nausea [40], which may worsen the TKI-induced AEs and likely affect the quality of life.

However, mild and moderate hypertension, which is usually asymptomatic, does not reduce the quality of life; patients with hypertension grade 2 or higher have a higher risk for CVD [58,59,63]. It is important to remember that TKI treatment is a life-prolonging therapy and not a cure (25, 36). The 10-year survival rate drops from 95.6% without any metastases to 40% when metastases have been diagnosed [78], and hypertensive consequences do not have the time to develop and, therefore, may be of less importance for patients suffering from progressive MTC compared to their cancer activity.

No direct comparison between the two studies of vandetanib can be made regarding efficiency due to different kinds of studies. A 100 mg/day vandetanib treatment was investigated through a single-arm study used to collect more data but not to confirm or conclude on efficiency [79].

It can be argued, though, that a smaller dose also entails a lower anticancer response rate. Results from a study investigating vandetanib showed that 23% of the patients experienced progression in disease despite an intake of 150 mg vandetanib, and only 5% experienced progression within the patients receiving 300 mg vandetanib [80]. The balance of CVD risk and anticancer treatment should favor anticancer treatment when the survival rate against cancer is low.

## 4. Methods

Publications were collected from the PubMed database and clinical trials; the last search was on 11 November 2022. To narrow down the information concerning the safety and/or efficiency of TKIs following keywords were used for the literature search: “cabozantinib MTC” (125 results), “vandetanib MTC” (176 results), “selpercatinib MTC” (17 results), “pralsetinib MTC” (11 results), “vandetanib, cabozantinib, pralsetinib, selpercatinib” (17 results), “Multikinase inhibitors medullary, thyroid cancer” (78 results). Eligibility criteria included results published after January 1, 2011, with patients older than 18 years. Articles with a focus on the mechanisms of TKI, therapeutic use, advantages, and AEs with their role in TKI treatment were included. These searches gave a total of 396 results, with multiple articles acquired in more of the searches. Twenty articles met the inclusion criteria based on the abstract, publication date, and title.

## 5. Conclusions

Vandetanib, pralsetinib, cabozantinib, and selpercatinib are all TKIs showing significantly improved PFS and anticancer activity in patients suffering from progressed MTC. No curing results have yet been detected, and TKIs remain a life-prolonging treatment. Besides diarrhea, hypertension is a frequent and often serious AE and occurs mainly due to the VEGF targeting inducing a decreased NO and PGI2 expression and an increased ET-1 expression leading to hypertension following vandetanib and cabozantinib treatment. A similar hypertensive tendency is seen in the RET-selective TKIs due to unknown mechanisms. Hypertension is easy to diagnose and treat with first-line antihypertensive drugs, such as ACE inhibitors, ARBs, or BAAs. Hypertension is important to control in order to prevent CVD, a dose reduction, or discontinuation of the TKI. However, the hypertensive AE seems to be of less importance and is outweighed by other, more difficult-to-manage AEs. The latest results favor the RET-selective TKIs as first-line treatment, but this is not a widely recognized opinion due to a lack of data. This conclusion is based on the greater anticancer activity and fewer changes in the initiated therapy compared to the conventional treatment regimen with cabozantinib and vandetanib. Since the percentage of hypertensive cases of a higher grade was superior among the RET-selective drugs, this must be ascribed to other AEs like diarrhea, rash, and nausea. In 2020, the FDA approved pralsetinib and selpercatinib; therefore, further data are needed.

## Figures and Tables

**Table 1:** Overview of the four drugs and their respective targets and approval for use in the treatment of MTC.

Drug	Targets	Dose	Approval for MTC	Structure
Cabozantinib [28,29]	MET, RET and VEGFR-2	140 mg/day	2012 (FDA) 2013 (EMA	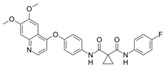
Vandetanib [30,31]	RET, VEGFR-2 and EGFR	300 mg/day	2012 (FDA) 2012 (EMA)	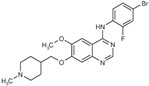
Selpercatinib [32]	RET	160 mg twice a day	2020 (FDA) 2021 (EMA)	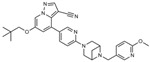
Pralsetinib [26]	RET	300mg/day	2020 (FDA) NA (EMA)	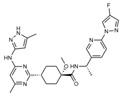

Only receptors inhibited by cabozantinib, which are being investigated in the EXAM study, are mentioned. FDA: Food and Drug Administration; EMA European Medicines Agency; NA: not applicable.

**Table 2:** The categories of hypertension and their corresponding range of BP.

Category	Systolic (mmHg)	Diastolic (mmHg)
Optimal	<120	<80
Normal	120–129	80–84
High normal	130–139	85–89
Grade 1 hypertension	140–159	90–99
Grade 2 hypertension	160–170	100–109
Grade 3 hypertension	≥180	≥110

Adapted from the ESC/ESH guidelines [34]; BP: Blood pressure. Accounts for all ages above 15 years.

**Table 3:** Overview of the discussed clinical trials and their outcomes.

Study	Deign	Participants	Outcome
Cabozantinib vs. placebo (EXAM) (NCT00704730) [28,56]	Phase III	219 vs. 111	PFS of 11.2 months vs. 4.0 months HR: 0.28;95%CI: 0.19–0.40; *p* < 0.001
Vandetanib vs. placebo (ZETA) (NCT00410761) [30,63]	Phase III	231 vs. 100	PFS of 30.5 months vs. 19.3 months HR: 0.46; 95%CI: 0.31–0.69; *p* < 0.001
Selpercatinib (LIBRETTO-001) (NCT04280081) [25]	Phase I/II	55 pretreated 88 untreated All RET-mutated	HR: 0.73; 95%CI: 0.62–0.82
Pralsetinib (ARROW) (NCT03037385) [26,55]	Phase I/II	55 pretreated 29 untreated All RET-mutated	HR: 0.71; 95%CI: 0.48–0.89

vs = versus, ORR = Objective response rate, PFS = progression-free survival.

**Table 4:** Occurrence of the AE hypertension and dose modifications because of (all) AEs.

	Grade 3–4	Dose Reduction	Interruption	Discontinuation
Selpercatinib [25]	21%	31%	5%	2%
Pralsetinib [26]	17%	NA	NA	4%
Cabozantinib [28]	8.4%	79%	65%	16%
Vandetanib [30]	9%	35%	NA	12%

NA—not available.

**Table 5:** Top five most common AEs, any grade, for each drug (%).

	Selpercatinib [25]		Pralsetinib [55]		Vandetanib [30]		Cabozantinib [28]	
Most common AEs	Dry mouth	39	Musculoskeletal pain	42	Diarrhea	56	Diarrhea	63
	Diarrhea	37	Constipation	41	Rash	45	Palmar-plantar Erythrodysesthesia	50
	Hypertension	35	Hypertension	40	Nausea	33	Decreased weight	48
	Fatigue	35	Fatigue	38	Hypertension	32	Decreased appetite	48
	Oedema	33	Diarrhea	34	Headache	26	Nausea	43

**Table 6:** Currently active clinical trials for the use of pralsetinib, cabozantinib, vandetanib, or selpercatinib.

Title and NCT Number	Design	Recruitment Status
A phase III, randomized, open-label study of pralsetinib versus standard of care for treatment of RET-mutated medullary thyroid cancer (NCT04760288)	A phase III, randomized, open-label study of pralsetinib versus standard of care for treatment of RET-mutated medullary thyroid cancer	Not yet recruiting
A phase 1/2 study of the highly-selective RET inhibitor, BLU-667, in patients with thyroid cancer, non-small cell lung cancer (NSCLC), and other advanced solid tumors (NCT03037385)	A phase I/II, non- randomized, open-label, first-in-human study	Active, not recruiting
A multicenter, randomized, open-label, phase 3 trial comparing selpercatinib to physicians’ choice of cabozantinib or vandetanib in patients with progressive, advanced, kinase inhibitor naive, RET-mutant medullary thyroid cancer (LIBRETTO-531) (NCT04211337)	A phase III, randomized, open-label study	Recruiting
A phase 1/2 study of oral selpercatinib (LOXO-292) in patients with advanced solid tumors, including RET fusion-positive solid tumors, medullary thyroid cancer, and other tumors with RET activation (LIBRETTO-001) (NCT03157128)	A phase I/II, open-label study	Recruiting
Neoadjuvant treatment with selpercatinib in RET-altered thyroid cancers cancer (NCT04759911)	A phase II, open-label study	Recruiting
An international, randomized, double-blind, two-arm study to evaluate the safety and efficacy of vandetanib 150 And 300mg/Day In Patients With Unresectable Locally Advanced Or Metastatic Medullary Thyroid Carcinoma With Progressive Or Symptomatic Disease (NCT01496313)	A phase IV, randomized, double-blind study	Active, not recruiting
A randomized, double-blind study to evaluate the efficacy and safety of cabozantinib (XL184) at 60 mg/day compared to a 140 mg/day in progressive, metastatic medullary thyroid cancer patients (NCT01896479)	A phase IV, randomized, double-blind study	Active, not recruiting
A multicenter, randomized, open-label, phase 3 trial comparing selpercatinib to physicians’ choice of cabozantinib or vandetanib in patients with progressive, advanced, kinase inhibitor naïve, RET-mutant medullary thyroid cancer (LIBRETTO-531) (NCT04211337)	A phase III, randomized, open-label study	Recruiting
Pilot trial of nivolumab plus cabozantinib for advanced solid tumors in patients with HIV infection (NCT04514484)	A phase I, open-label study	Recruiting
A Randomized, Int., Open-Label Phase III Study to Assess the Effect of a Patient Outreach Program on the Percentage of Time Patients With Locally Advanced or Metastatic MTC Experience Grade 2 or Higher AEs in the First 12 Months of Treatment With Vandetanib (NCT01298323)	A phase III, open-label study	Active, not recruiting
Effects of tyrosine kinase inhibitors on body composition in endocrine tumors—a pilot study (NCT02592356)	Phase is not applicable, open-label study	Active, not recruiting

Active, not recruiting studies are included if the ‘estimated study complete’ date is not expired.

**Table 7:** Completed clinical trials for the use of pralsetinib, cabozantinib, vandetanib, or selpercatinib.

Title and NCT Number	Design	Recruitment Status
An international, randomized, double-blinded, phase 3 efficacy study of XL184 versus placebo in subjects with unresectable, locally advanced, or metastatic medullary thyroid cancer (NCT00704730)	A phase III, randomized, double-blinded study	Completed
Molecular profile of metastatic sporadic medullary thyroid cancer (sMTC) patients and possible correlation with vandetanib therapy (NCT02268734)	Observational study	Completed
European, observational, prospective study to evaluate the benefit/risk of vandetanib in RET mutation negative and positive patients with symptomatic, aggressive, sporadic, unresectable, locally advanced/metastatic medullary thyroid cancer (NCT01945762)	Observational study	Completed
A phase I/II, open-label study to evaluate the safety and tolerability of vandetanib 300 mg/Day in Japanese patients With unresectable locally advanced or metastatic medullary thyroid carcinoma (NCT01661179)	A phase I/II, open-label study	Completed
CAPRELSA^®®^ REGISTRY: a Belgian registry to evaluate the use of vandetanib (Caprelsa^®®^) in current clinical practice (NCT02109250)	Observational study	Completed
Effectiveness of Risk minimization interventions for vandetanib in Canada (NCT01757470)	Observational study	Completed
A Phase I, randomized, open-label, single-center study to assess the pharmacokinetics of vandetanib (CAPRELSA) in healthy subjects when a single oral dose of vandetanib 300 mg is administered alone and in combination with omeprazole or ranitidine (NCT01539655)	A phase I, randomized, open-label study	Completed

Only accounting studies on adults.

**Table 8:** Recommended drugs for TKI-induced hypertension.

**Recommended antihypertensive drugs**	ACE inhibitors ARB BAA CCB (but not in combination with cabozantinib)

ACE: angiotensin-converting enzyme. ARB: angiotensin receptor blocker. BAA: beta-adrenoceptor antagonist. CCB: calcium channel blocker.

## Data Availability

Not applicable.

## Abbreviations

AE	adverse effect
ACE	angiotensin-converting enzyme
AKT	protein kinase B
ARB	angiotensin receptor blocker
AXL	tyrosine-protein kinase receptor UFO
BAA	beta-adrenoceptor antagonist
BAD	BCL2 associated agonist of cell death
BP	blood pressure
CCB	calcium channel blocker
CVD	cardiovascular disease
CO	cardiac output
EFGR	epidermal growth factor receptor
ERK	extracellular regulated kinase
ET-1	endothelin-1
FDA	food and drug administration
FLT-3	fms-like tyrosine kinase 3
GF	growth factor
HGF	hepatocyte growth factor
HR	hazard ration
KIT	tyrosine-protein kinase KIT or CD117
MAP	mean arterial pressure
MAPK	mitogen-activated protein kinase
MDM2	mouse double minute 2
MEK	mitogen-activated protein kinase kinase-1
MET	mesenchymal-epithelial transition
MET	tyrosine-protein kinase Met or hepatocyte growth factor receptor
TKI	multi-kinase inhibitor
MTC	medullary thyroid cancer
mTOR	mammalian target of rapamycin
NO	nitric oxide
NTRK-2	neurotrophic tyrosine receptor kinase 2
OS	overall survival
PDGF	platelet-derived growth factor
PGI2	prostaglandins
PI3K	phosphoinositide 3 kinases
PFS	progression-free survival
RAF	rapidly accelerated fibrosarcoma
RAS	rat sarcoma
RECIST	Response Evaluation Criteria in Solid Tumors
RET	rearranged during transfection
RTK	receptor tyrosine kinase
TC	thyroid cancer
TIE-2	angiopoietin-1 receptor
TRKB	tropomyosin receptor kinase B
TRP	total peripheral resistance
VEGF	vascular endothelial growth factor
VEGFR	vascular endothelial growth factor receptor
VS	versus
